# LEVERAGING NEIGHBORHOOD EXPOSURES AS MODIFIERS OF INDIVIDUAL RISK FACTORS FOR COGNITIVE DECLINE AND DEMENTIAS

**DOI:** 10.1093/geroni/igad104.1185

**Published:** 2023-12-21

**Authors:** Michelle Carlson, Andrea Rosso, Gina Lovasi

## Abstract

There are numerous ways in which neighborhood environmental factors have been shown to contribute to adverse health. More remains to be done to link long-term neighborhood exposures, such as social and physical activities, to modifiable individual risk factors for cognitive decline and dementia. Individual interventions are usually small in scale and conducted in those with higher socioeconomic status (SES). Additionally, increases in healthy behaviors, such as walking, have been difficult to promote and sustain. Each of these risk factors is known to be influenced by environmental features. This symposium will explore the roles of neighborhood-level social (nSES, affluence, cohesion, discrimination) and physical (air pollution) factors as drivers of cognitive aging and dementia across multiple longitudinal studies of community-based aging. Studies span urban and rural communities, including the Health ABC Study, the Cardiovascular Health Study, and the Think Phresh study. These studies are important to evaluating the role of neighborhood features on cognitive aging by expanding beyond cross-sectional data and by studying residentially stable older adults most likely to be affected by their local context. Those neighborhood factors that impact individual activity, cognitive aging, and Alzheimer’s disease risk will be further discussed for differences by race/ethnicity.

